# Supplementary material for: Role and mechanism of NCAPD3 in promoting malignant behaviors in gastric cancer
Source: Front Pharmacol. 2024 Apr 22;15:1341039. doi: 10.3389/fphar.2024.1341039 (PMC11070777; doi:10.3389/fphar.2024.1341039)
Supplement: Supplementary file 11 [file DataSheet2.ZIP › GSEA/GO_CC/my_analysis.Gsea.1599461408869/GO_SECRETORY_GRANULE.html]

Details for gene set GO\_SECRETORY\_GRANULE[GSEA]

|  || Dataset | filtered\_dataset.sample\_info.cls#WT\_versus\_NCAPD3\_MUT |
| Phenotype | sample\_info.cls#WT\_versus\_NCAPD3\_MUT |
| Upregulated in class | NCAPD3\_MUT |
| GeneSet | GO\_SECRETORY\_GRANULE |
| Enrichment Score (ES) | -0.23271507 |
| Normalized Enrichment Score (NES) | -1.5705062 |
| Nominal p-value | 0.025641026 |
| FDR q-value | 0.12147281 |
| FWER p-Value | 0.81 |
Table: GSEA Results Summary

  

Fig 1: Enrichment plot: GO\_SECRETORY\_GRANULE      
 Profile of the Running ES Score & Positions of GeneSet Members on the Rank Ordered List

  

| SYMBOL | TITLE | RANK IN GENE LIST | RANK METRIC SCORE | RUNNING ES | CORE ENRICHMENT || 1 | 2243 | FGA | 18 | 1.039 | 0.0163 | No |
| 2 | 5099 | PCDH7 | 23 | 1.026 | 0.0426 | No |
| 3 | 5967 | REG1A | 100 | 0.788 | 0.0087 | No |
| 4 | 3303 | HSPA1A | 206 | 0.669 | -0.0501 | No |
| 5 | 3304 | HSPA1B | 207 | 0.669 | -0.0310 | No |
| 6 | 81619 | TSPAN14 | 212 | 0.666 | -0.0150 | No |
| 7 | 5701 | PSMC2 | 244 | 0.636 | -0.0199 | No |
| 8 | 353376 | TICAM2 | 271 | 0.617 | -0.0216 | No |
| 9 | 10585 | POMT1 | 284 | 0.606 | -0.0132 | No |
| 10 | 538 | ATP7A | 305 | 0.588 | -0.0112 | No |
| 11 | 25852 | ARMC8 | 307 | 0.588 | 0.0048 | No |
| 12 | 2244 | FGB | 321 | 0.582 | 0.0117 | No |
| 13 | 960 | CD44 | 339 | 0.573 | 0.0155 | No |
| 14 | 5036 | PA2G4 | 365 | 0.557 | 0.0128 | No |
| 15 | 5718 | PSMD12 | 372 | 0.552 | 0.0241 | No |
| 16 | 387263 | C6orf120 | 400 | 0.535 | 0.0193 | No |
| 17 | 2919 | CXCL1 | 405 | 0.534 | 0.0316 | No |
| 18 | 1755 | DMBT1 | 480 | 0.487 | -0.0094 | No |
| 19 | 3479 | IGF1 | 511 | 0.469 | -0.0183 | No |
| 20 | 23250 | ATP11A | 664 | 0.395 | -0.1198 | No |
| 21 | 178 | AGL | 711 | 0.368 | -0.1434 | No |
| 22 | 203068 | TUBB | 736 | 0.358 | -0.1510 | No |
| 23 | 25825 | BACE2 | 756 | 0.344 | -0.1552 | No |
| 24 | 23197 | FAF2 | 760 | 0.342 | -0.1477 | No |
| 25 | 9749 | PHACTR2 | 806 | 0.319 | -0.1720 | No |
| 26 | 8904 | CPNE1 | 814 | 0.308 | -0.1684 | No |
| 27 | 3146 | HMGB1 | 831 | 0.295 | -0.1718 | No |
| 28 | 3312 | HSPA8 | 848 | 0.244 | -0.1768 | No |
| 29 | 6515 | SLC2A3 | 873 | -0.283 | -0.1865 | No |
| 30 | 23412 | COMMD3 | 894 | -0.312 | -0.1924 | No |
| 31 | 4973 | OLR1 | 944 | -0.366 | -0.2184 | No |
| 32 | 5329 | PLAUR | 952 | -0.373 | -0.2129 | No |
| 33 | 4680 | CEACAM6 | 955 | -0.374 | -0.2037 | No |
| 34 | 55591 | VEZT | 973 | -0.389 | -0.2053 | No |
| 35 | 6286 | S100P | 1011 | -0.412 | -0.2210 | Yes |
| 36 | 51646 | YPEL5 | 1014 | -0.413 | -0.2107 | Yes |
| 37 | 978 | CDA | 1023 | -0.421 | -0.2046 | Yes |
| 38 | 966 | CD59 | 1040 | -0.436 | -0.2040 | Yes |
| 39 | 54676 | GTPBP2 | 1050 | -0.447 | -0.1979 | Yes |
| 40 | 1191 | CLU | 1056 | -0.450 | -0.1888 | Yes |
| 41 | 6590 | SLPI | 1062 | -0.453 | -0.1796 | Yes |
| 42 | 1604 | CD55 | 1090 | -0.468 | -0.1863 | Yes |
| 43 | 1889 | ECE1 | 1091 | -0.468 | -0.1729 | Yes |
| 44 | 5834 | PYGB | 1096 | -0.475 | -0.1624 | Yes |
| 45 | 857 | CAV1 | 1098 | -0.475 | -0.1495 | Yes |
| 46 | 6709 | SPTAN1 | 1114 | -0.487 | -0.1468 | Yes |
| 47 | 6947 | TCN1 | 1135 | -0.503 | -0.1473 | Yes |
| 48 | 27074 | LAMP3 | 1158 | -0.522 | -0.1487 | Yes |
| 49 | 5154 | PDGFA | 1191 | -0.566 | -0.1563 | Yes |
| 50 | 5795 | PTPRJ | 1210 | -0.586 | -0.1529 | Yes |
| 51 | 22931 | RAB18 | 1231 | -0.608 | -0.1504 | Yes |
| 52 | 7057 | THBS1 | 1242 | -0.620 | -0.1402 | Yes |
| 53 | 1893 | ECM1 | 1250 | -0.637 | -0.1272 | Yes |
| 54 | 11057 | ABHD2 | 1258 | -0.646 | -0.1140 | Yes |
| 55 | 55 | ACPP | 1265 | -0.656 | -0.0997 | Yes |
| 56 | 7414 | VCL | 1308 | -0.712 | -0.1106 | Yes |
| 57 | 54742 | LY6K | 1331 | -0.755 | -0.1053 | Yes |
| 58 | 55521 | TRIM36 | 1337 | -0.774 | -0.0870 | Yes |
| 59 | 1116 | CHI3L1 | 1339 | -0.777 | -0.0656 | Yes |
| 60 | 7422 | VEGFA | 1346 | -0.788 | -0.0475 | Yes |
| 61 | 2040 | STOM | 1389 | -0.988 | -0.0505 | Yes |
| 62 | 2444 | FRK | 1396 | -1.082 | -0.0241 | Yes |
| 63 | 3329 | HSPD1 | 1402 | -1.183 | 0.0059 | Yes |
Table: GSEA details [plain text format]

  

Fig 2: GO\_SECRETORY\_GRANULE      
 Blue-Pink O' Gram in the Space of the Analyzed GeneSet

  

Fig 3: GO\_SECRETORY\_GRANULE: Random ES distribution      
 Gene set null distribution of ES for **GO\_SECRETORY\_GRANULE**
